# Supplementary material for: MAIT cell responses to Staphylococcus aureus and sensitivity to HlgAB toxin are modulated by activation and tissue-dependent virulence effects
Source: mBio. 2026 Sep 17;17(10):e00663-26. doi: 10.1128/mbio.00663-26 (PMC13713009; doi:10.1128/mbio.00663-26)
Supplement: Supplementary file 1 [file mbio.00663-26-s0001.pdf]

Supplementary material (methods, four tables and three figures) to the manuscript:

MAIT cell responses to *Staphylococcus aureus* and sensitivity to HlgAB toxin are modulated by activation and tissue-dependent virulence effects

Elisa J. M. Raineri[1], Caroline Boulouis[1*], Elli Mouchtaridi[1*], Vera Nilsén[1,2,3], Curtis Cai[1], Tobias Kammann[1], Juliette Tabusse[1], Takuya Sekine[1], Nicole Wild[4], Christian Constantz[1], Eoghann White[1], Thomas R. Müller[1], Anne Marchalot[1], Sabrina Ferreira[1], Jyotsana Kaushal[1], Akhirunnesa Mily[1], Miriam Franklin[1], Elena Bonaiti[4], Mary-Lyn Eichhorn[4], John Bassett[1], Chris Stamper[1], Jeffrey Y. W. Mak[5], David P. Fairlie[5], Chris Tibbitt[1], Anna Norrby-Teglund[1], Nicole Marquardt[4], Jenny Mjösberg[1], Carl Jorns[2,3], Jenny Driving[6], Edwin Leeansyah[1,7,8], Marcus Buggert[1] and Johan K. Sandberg[1,#]

[1]Center for Infectious Medicine, Department of Medicine, Karolinska Institutet, Stockholm, Sweden.
[2]ME Transplantation, Karolinska University Hospital Huddinge, Stockholm, Sweden.
[3]Department of Clinical Science, Intervention and Technology, Karolinska Institutet, Stockholm, Sweden.
[4]Center for Hematology and Regenerative Medicine, Department of Medicine Huddinge, Karolinska Institutet, Stockholm, Sweden.
[5]Centre for Chemistry and Drug Discovery, Australian Research Council Centre of Excellence for Innovations in Peptide and Protein Science, Institute for Molecular Bioscience, The University of Queensland, Brisbane, Australia.
[6]Capio Öron Näsa Hals Globen/Odenplan, Stockholm, Sweden.
[7]Centre for Innate Immunity and Infectious Diseases, Hudson Institute of Medical Research, Clayton, Victoria, Australia.
[8]Department of Molecular and Translational Science, Monash University, Clayton, Victoria, Australia.

[#]**Correspondence:** Dr. Johan K. Sandberg, Center for Infectious Medicine, Department of Medicine, Karolinska Institutet, 14152 Stockholm, Sweden. E-mail: johan.sandberg@ki.se

Supplementary materials and methods

Preparation of cells from tissues and blood

Peripheral blood mononuclear cells (PBMCs) were isolated using Ficoll-Paque Plus (Sigma-Aldrich). Following isolation, PBMCs were incubated overnight in RPMI-1640 medium supplemented with 25 mM HEPES, 2 mM L-glutamine (Thermo Fisher Scientific), 10% fetal bovine serum (Sigma-Aldrich), 50 μg/ml gentamicin (Life Technologies), and 100 ml normocin (InvivoGen), referred to as R10 complete medium. For selected experiments, Vα7.2+ cells were isolated from PBMCs using an anti-Vα7.2 PE-Cy7 conjugated monoclonal antibody (3C10, BioLegend) and positive selection with magnetic-activated cell sorting (MACS) using anti-PE microbeads (Miltenyi Biotec), according to the manufacturer's instructions.

Human organ donor tissues were transported immediately after surgery, rinsed with PBS, and processed in sterile petri dishes containing RPMI 1640 (Gibco) supplemented with 10% heat-inactivated FCS (MilliporeSigma), 1 mM L-glutamine (Invitrogen), 100 U/ml penicillin, 50 μg/ml streptomycin, and 1 nM DNase I (Sigma-Aldrich). Lung parenchyma and lung-associated lymph nodes were dissected separately; mesenteric lymph nodes and gut segments (ileum, cecum, colon) were processed individually. Gut tissues were washed with calcium/magnesium-free HBSS (Thermo Fisher) containing gentamycin (10 mg/ml), penicillin, streptomycin (Cytiva), and fungizone (250 μg/ml). After removing adipose and muscle, all tissues were manually dissected and digested in RPMI 1640 with DNase I, and collagenase (80 μg/ml; Worthington) was added to intestinal specimens only, for 45-60 min at 37°C. Post-incubation, tissues were strained and resuspended in R10; spleen and lung suspensions underwent red cell lysis (Invitrogen). PBMCs from organ donors were isolated by Ficoll-Paque Plus (Sigma-Aldrich) density gradient centrifugation and resuspended in R10. Cells were plated at 2×10^6 per well in 96-well U-bottom plates (Corning) and used for fresh staining.

Tonsils were cleaned, minced, ground, and filtered through 100 μm and then 70 μm cell strainers. The resulting cell suspension was washed with PBS, and mononuclear cells were cryopreserved in FBS (Thermo Fisher Scientific) containing 10% DMSO (Sigma-Aldrich) or plated for fresh staining. To enable comparison between blood and tonsil cells, cryopreserved samples were thawed and rested for 3 h before staining (2×10^6 cells per well in 96-well U bottom plates (Corning)). Blood samples used in these comparisons were initially processed by Ficoll-Paque Plus (Sigma-Aldrich) prior to freezing.

Analysis of flow cytometry data by COMPASS

COMPASS (Combinatorial Polyfunctionality Analysis of Antigen-Specific T cell Subsets) is a Bayesian framework that models the full space of possible effector marker combinations and estimates, for each combination, the posterior probability that it is enriched in stimulated compared to unstimulated conditions. For each donor, a functionality score was derived to summarize the overall polyfunctional response, and richness was defined as the number of effector combinations with a posterior probability > 0.01. A heatmap was used to visualize the mean posterior probability of response across all functional combinations. The Simple COMPASS function was applied to MAIT cells in two complementary analyses: (i) a dose-response analysis at 24 h comparing the three bacterial doses MOI 50, MOI 100 and MOI 500 against unstimulated control cells, and (ii) a time course analysis using MOI 100 across five time points against unstimulated control cells at each time point. The marker panel included CD107a, granzyme B, IL-10, IFNγ, perforin and TNF. Per-donor boolean gate counts for each marker combination were exported from FlowJo and used as input to the COMPASS package in R (v1.3.0), run with eight replications of 40,000 MCMC iterations. To ensure equal contribution of each sample to the model, all donors were normalized to the lowest cell count observed across donors and conditions; for donors exceeding this threshold, marker combination counts were resampled proportionally to preserve the original distribution. Statistical comparisons of functionality score and richness were performed using the Wilcoxon matched-pairs signed-rank test with Benjamini-Hochberg correction for multiple comparisons.

Supplementary Table 1. Donor characteristics of the organ donors (IHOPE cohort)

Donor	Age	Sex	CMV	EBV
IHOPE36	43	Female	Negative	Positive
IHOPE37	63	Female	Negative	Positive
IHOPE38	32	Female	Positive	Positive
IHOPE39	68	Male	Positive	ND
IHOPE47	62	Male	Positive	Positive
IHOPE50	71	Female	Positive	Positive
IHOPE51	74	Female	Positive	ND
IHOPE52	56	Female	Negative	Positive
IHOPE53	53	Male	Positive	ND

ND=Not done

Supplementary Table 2. Donor characteristics of the tonsils (frozen samples).

Donor	Tonsils	PBMCs	Age	Sex	Reason of Surgery	CMV	EBV
TONCIM 22	+		18	Female	RT	ND	ND
TONCIM 51	+		22	Female	P	ND	ND
TONCIM 99	+		19	Male	CT	ND	ND
TONCIM 100	+		41	Male	RT	ND	ND
TONCIM 113	+		31	Male	OSAS	ND	ND
TONCIM 126	+		48	Male	RT	ND	ND
TONCIM 140	+		18	Male	RT	ND	ND
TONCIM 145	+		16	Female	P	ND	ND
TONCIM 147	+		22	Male	CT	ND	ND
TONCIM 148	+		19	Male	CT	ND	ND
TONCIM 162	+		23	Female	RT	ND	ND
TONCIM 167	+		31	Female	RT	ND	ND
TONCIM 182	+	+	18	Male	CT	ND	ND
TONCIM 187	+	+	24	Female	RT	ND	ND
TONCIM 200	+		26	Male	RT	ND	ND
TONCIM 202	+		30	Female	CT	ND	ND
TONCIM 205	+	+	34	Female	RT	ND	ND
TONCIM 207	+		21	Female	CT	ND	ND
TONCIM 209	+	+	26	Male	CT	ND	ND
TONCIM 212	+		27	Female	CT	ND	ND
TONCIM 216	+	+	37	Female	CT	ND	ND
TONCIM 221	+	+	24	Female	CT	ND	ND
TONCIM 223	+		20	Male	CT	ND	ND
TONCIM 229	+	+	30	Female	P	ND	ND
TONCIM 248	+		48	Male	P	ND	ND
TONCIM 251	+		19	Male	P	ND	ND
TONCIM 256	+		26	Male	OSAS	ND	ND
TONCIM 263	+		55	Male	OSAS	ND	ND
TONCIM 269	+		31	Female	OSAS	ND	ND
TONCIM 273	+		39	Male	P	ND	ND
TONCIM 291	+		16	Male	OSAS	ND	ND
TONCIM 317	+		34	Male	OSAS	ND	ND

ND=Not done
RT=Recurrent tonsillitis
OSAS=Obstructive sleep apnea syndrome
P=Peritonsillitis
CT=Chronic tonsillitis

Supplementary Table 3. Donor characteristics of the tonsils (fresh samples)

Donor	Tonsils	Age	Sex	Reason of Surgery	CMV	EBV
TONCIM 248	+	48	Male	P	ND	ND
TONCIM 249	+	12	Male	CT	ND	ND
TONCIM 250	+	18	Female	CT	ND	ND
TONCIM 251	+	19	Male	P	ND	ND
TONCIM 260	+	21	Female	RT	ND	ND
TONCIM 261	+	17	Female	RT	ND	ND
TONCIM 262	+	21	Female	CT	ND	ND
TONCIM 263	+	55	Male	OSAS	ND	ND
TONCIM 269	+	31	Female	OSAS	ND	ND
TONCIM 270	+	30	Male	CT	ND	ND
TONCIM 278	+	27	Male	RT	ND	ND
TONCIM 279	+	19	Male	RT	ND	ND
TONCIM 280	+	20	Female	RT	ND	ND
TONCIM 281	+	22	Female	RT	ND	ND

ND=Not done
RT=Recurrent tonsillitis
OSAS=Obstructive sleep apnea syndrome
P=Peritonsillitis
CT=Chronic tonsillitis

Supplementary Table 4. List of reagents used for flow cytometry staining.

Antigen	Fluorochrome	Clone	Provider
CD3	AF700	UCHT1	BD Biosciences
CD3	BUV805	SK7	BD Biosciences
CD8	BUV395	SK7	BD Biosciences
CD8	BV570	RPA-T8	BioLegend
CD4	BV711	OKT4	BioLegend
Vα7.2	PE-Cy7	3C10	BioLegend
CD14	BUV395	M5E2	BD Biosciences
CD14	BV750	M5E2	BD Biosciences
CD161	PE-Cy5	DX10	BD Biosciences
CD69	BUV615	FN50	BD Biosciences
CD69	BUV650	FN50	BD Biosciences
CD107a	BUV395	H4A3	BD Biosciences
CD56	BUV737	NCAM16.2	BD Biosciences
CD56	BUV661	NCAM16.2	BD Biosciences
CD45	BUV805	H130	BD Biosciences
HLA-DR	BUV563	G46-6	BD Biosciences
CD38	BV661	HIT2	BD Biosciences
CD25	BV711	BC96	BioLegend
Live/Dead Near IR	N/A	N/A	Invitrogen
Live/Dead AQUA	N/A	N/A	Invitrogen
5-OP-RU-hMR1	PE	N/A	NIH Tetramer Core Facility
5-OP-RU-hMR1	APC	N/A	NIH Tetramer Core Facility
CCR2	BV510	K036C2	BioLegend
CCR5	BV605	2D7	BD Biosciences
CXCR1	PE	8F1	BioLegend
CXCR2	BV785	6C6	BD Biosciences
IFNγ	BV785	4S.B3	BioLegend
IL-17A	BV421	BL168	BioLegend
IL-10	PE-CF594	9EJ3-19F1	BD Biosciences
IL-22	2G12A41	APC-Fire750	BioLegend
Granzyme B	FITC	GB11	BioLegend
Granzyme A	R718	CB9	BD Biosciences
Granzyme K	PE	G3H69	eBioscience
Granulysin	BB630	RB1	BD Biosciences
TNF	BV650	MAb11	BD Biosciences
Perforin	dG9	BB755	BD Biosciences

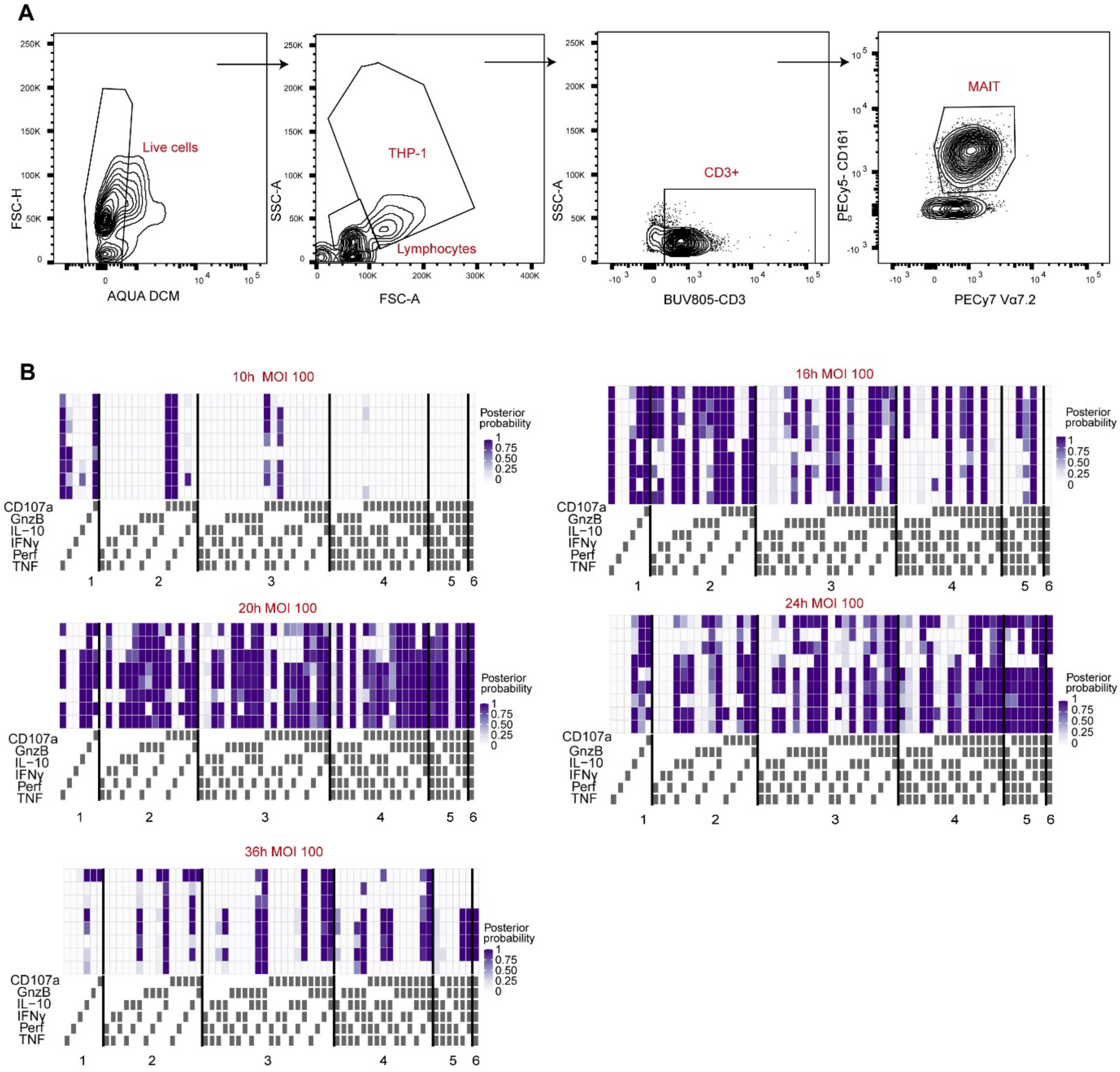

Supplementary Figure 1. MAIT cell gating and peripheral blood MAIT cell *S. aureus* response. (A) Gating strategy for identification of MAIT cells in peripheral blood and cocultured with THP-1 cells as APCs. (B) Analysis of polyfunctionality using the COMPASS algorithm to blood MAIT cells using MOI 100 across five time points (10 h, 16 h, 20 h, 24 h and 36 h) against unstimulated control cells at each time point. Rows of the heatmap show the MAIT cells and column indicate the combination of effector molecules in ascending degrees of polyfunctionality. Statistical comparison between conditions with the Wilcoxon matched-pairs signed-rank test. *p<0.05, **p<0.01, ***p<0.001 and ****p<0.0001.

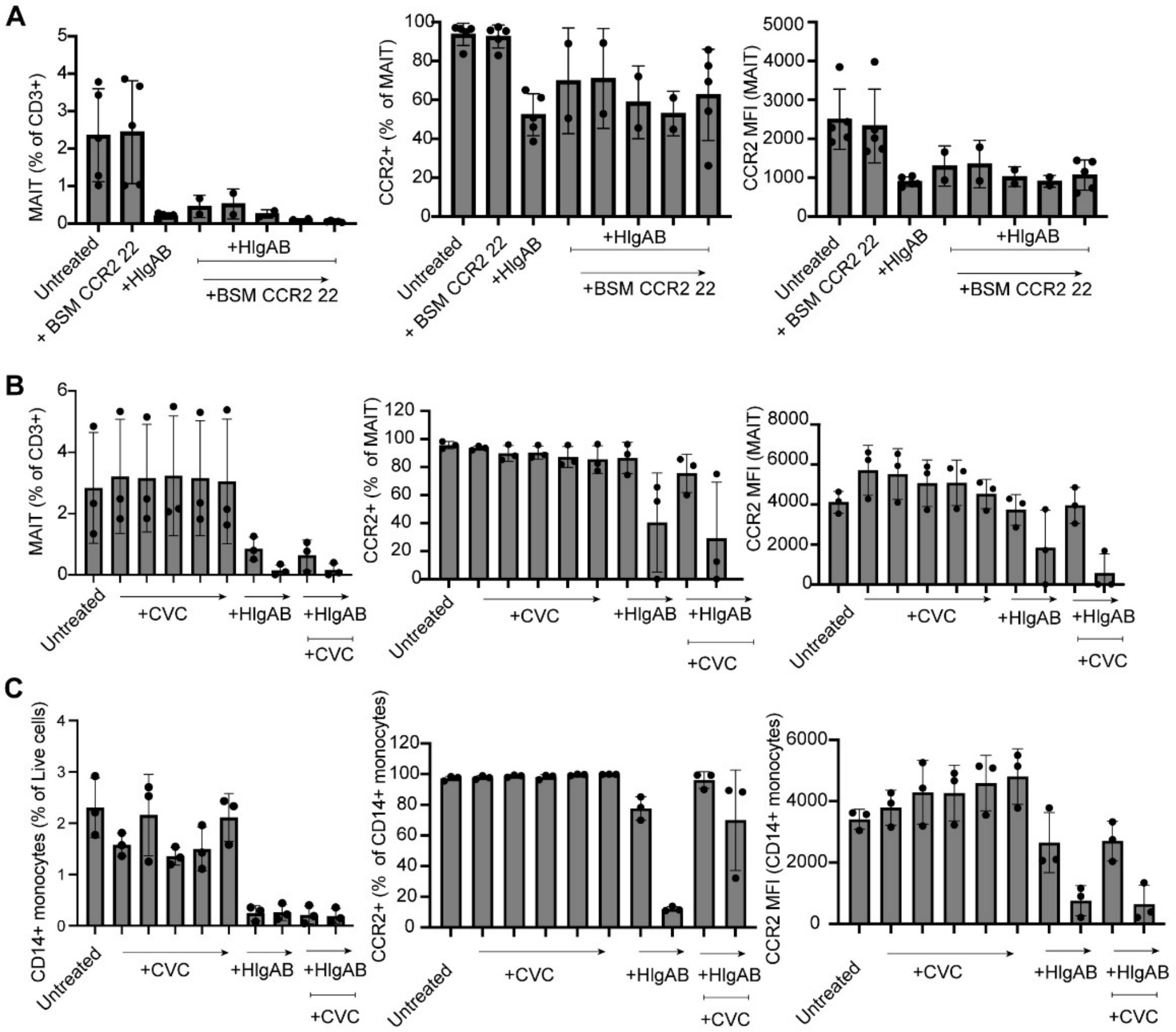

Supplementary Figure 2. CCR2 antagonists do not rescue MAIT cells from HIgAB toxicity in vitro. (A) MAIT cells frequencies and CCR2 expression after treatment with BSM CCR2 22 (n= 2-5). The CCR2 antagonist BSM CCR2 22 is used at 100 µM for 3 h and HIgAB was used at 10 µg/mL for the last 2 h. The arrow indicates increasing concentrations of CCR2 antagonist: 25 nM, 50 nM, 10 µM, 50 µM, and 100 µM. Percentages of MAIT cells (B) or CD14+ monocytes (C) and CCR2 expression after treatment with Cenicriviroc (CVC) (n= 3). The dual CCR2/CCR5 antagonist is used at 1 µM for 3 h and HIgAB was used at 2.5 and 0.31 µg/mL for the last 2 h. The arrow indicates increasing concentrations of CCR2 antagonist: 0.29 nM, 5.9 nM, 25 nM, 500 nM, and 1 µM.

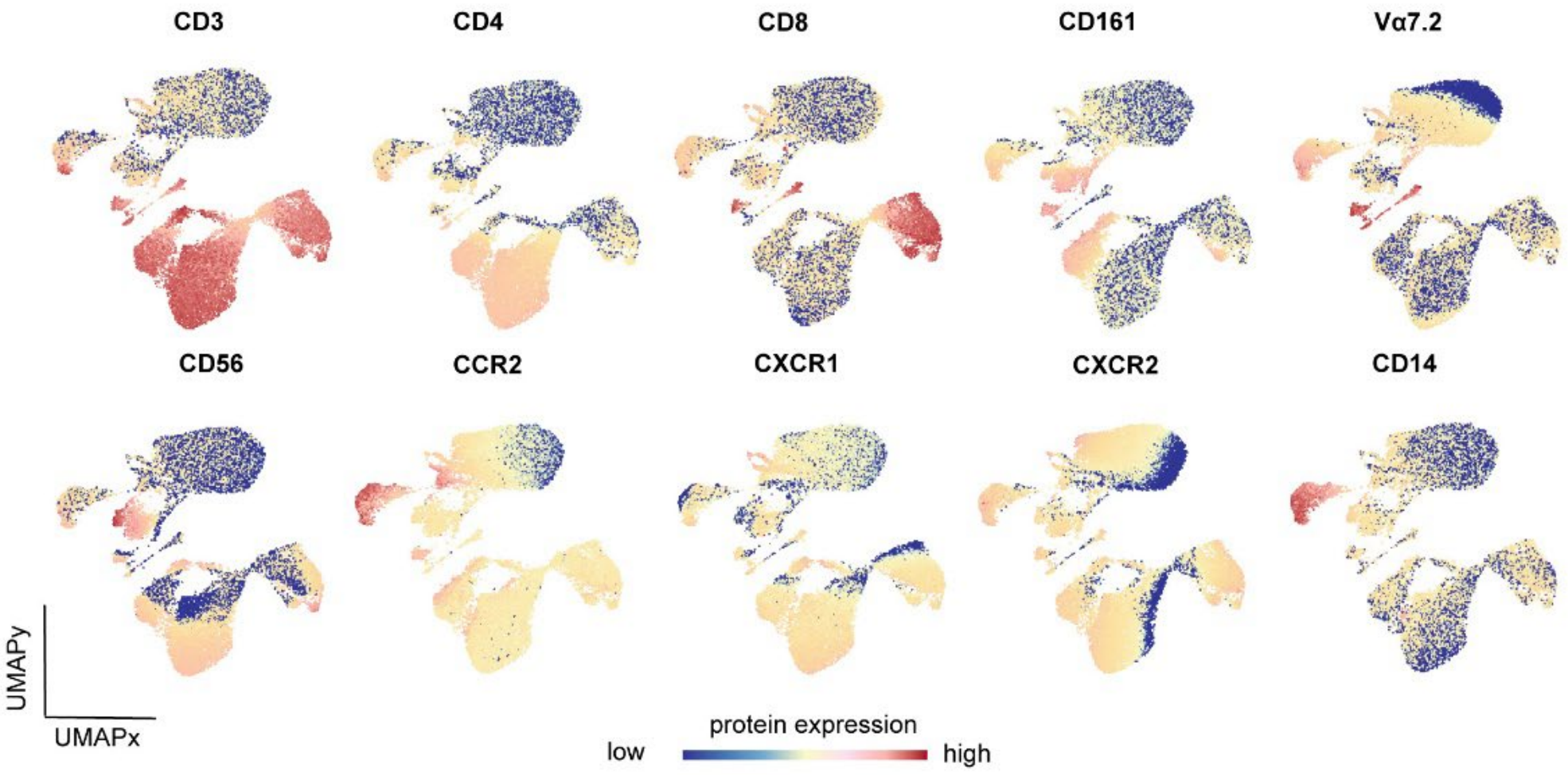

Supplementary Figure 3. Corresponding UMAP plots of total live PBMCs showing expression of the indicated markers (relating to Figure 4).
